# Whole-Genome Sequence Data Analysis of *Anoxybacillus kamchatkensis* NASTPD13 Isolated from Hot Spring of Myagdi, Nepal

**DOI:** 10.1155/2021/1869748

**Published:** 2021-06-27

**Authors:** Punam Yadav, Shikha Sharma, Tribikram Bhattarai, Lakshmaiah Sreerama, Gandham S. Prasad, Girish Sahni, Jyoti Maharjan

**Affiliations:** ^1^Molecular Biotechnology Unit, Nepal Academy of Science and Technology, Lalitpur, Nepal; ^2^Central Departments of Biotechnology, Tribhuvan University, Kirtipur, Nepal; ^3^Laboratory of Bacterial Genomics and Evolution, CSIR-Institute of Microbial Technology, Chandigarh, India; ^4^Department of Chemistry and Earth Sciences, Qatar University, Doha, Qatar; ^5^University of Hyderabad, Hyderabad, India; ^6^Institute of Microbial Technology, Chandigarh, India

## Abstract

*Anoxybacillus kamchatkensis* NASTPD13 isolated from Paudwar hot spring of Myagdi, Nepal, upon morphological and biochemical analysis revealed to be Gram-positive, straight or slightly curved, rod-shaped, spore-forming, catalase, and oxidase-positive facultative anaerobes. It grows over a wide range of pH (5.0-11) and temperature (37-75°C), which showed growth in different reduced carbon sources such as starch raffinose, glucose, fructose, inositol, trehalose, sorbitol, mellobiose, and mannitol in aerobic conditions. Furthermore, the partial sequence obtained upon sequencing showed 99% sequence similarity in 16S rRNA gene sequence with *A. kamchatkensis* JW/VK-KG4 and was suggested to be *Anoxybacillus kamchatkensis*. Moreover, whole-genome analysis of NASTPD13 revealed 2,866,796 bp genome with a G+C content of 41.6%. Analysis of the genome revealed the presence of 102 RNA genes, which includes sequences coding for 19 rRNA and 79 tRNA genes. While the 16S rRNA gene sequence of strain NASTPD13 showed high similarity (>99%) to those of *A. kamchatkensis* JW/VK-KG4, RAST analysis of NASTPD13 genome suggested that *A. kamchatkensis* G10 is actually the closest neighbor in terms of sequence similarity. The genome annotation by RAST revealed various genes encoding glycoside hydrolases supporting that it can utilize several reduced carbon sources as observed and these genes could be important for carbohydrate-related industries. Xylanase pathway, particularly the genomic region encoding key enzymes for xylan depolymerization and xylose metabolism, further confirmed the presence of the complete gene in xylan metabolism. In addition, the complete xylose utilization gene locus analysis of NASTPD13 genome revealed all including D-xylose transport ATP-binding protein XylG and XylF, the xylose isomerase encoding gene XylA, and the gene XylB coding for a xylulokinase supported the fact that the isolate contains a complete set of genes related to xylan degradation, pentose transport, and metabolism. The results of the present study suggest that the isolated *A. kamchatkensis* NASTPD13 containing xylanase-producing genes could be useful in lignocellulosic biomass-utilizing industries where pentose polymers could also be utilized along with the hexose polymers.

## 1. Introduction

*Anoxybacillus* was first described as a separate genus by Pikuta et al. [1] in contrast to *Bacillus* and *Geobacillus* based on their phenotypic properties, 16S rDNA sequence, and DNA–DNA hybridization experiments. They are defined as rod-shaped, present in pairs or short chains with terminal endospores, Gram-positive, aerotolerant or facultative anaerobe, thermophilic, and alkalophilic or alkalitolerant and grew at a temperature of 37-62°C [2]. Mostly, *Anoxybacillus* spp. have been reported from hot springs but it has also been isolated from different environments such as animal manure, dairy products, and guts of animals ([3]).

The only genome of *A. flavithermus* WK1 (PRJNA59135) [4] is completely sequenced, while the draft genomes of other species, including *A. ayderensis* AB04T (PRJNA258494), *A. thermarum* AF/04T (PRJNA260786), *A. gonensis* G2T (PRJNA264351), *A. tepidamans* PS2 (PRJNA214279), *A. kamchatkensis*G10 ((PRJNA170961), and 5 strains of *A. flavithermus*, 25 (PRJNA258119), Kn10 (PRJDB1085), TNO-09.006 (PRJNA169174), and E13T (PRJNA213809), and many more has been reported [5].

Several studies has reported regarding the importance of *Anoxybacillus* sp., and mostly, the enzymes produced by bacilli that can tolerate the harsh industrial conditions such as alkaline pH and high temperature[2]. Many researches are seeking an alternative for renewable energy generation [6]. Certain features of *Anoxybacillus* spp. such as able to grow fast, tolerating extreme conditions, and producing various thermozymes are getting attention of many researchers as it can resolve the issues related to biomass hydrolysis [7]. In addition, *Anoxybacillus* sp. genomes are relatively small in size and can be used as a microbial cell factory for minimizing the difficulty during genome engineering and also serve as a host for certain thermostable enzyme applications [8].

*Anoxybacillus kamchatkensis* have first been reported from Geyser valley located in Kamchatka peninsula [9]. In the current work, we describe the features of *Anoxybacillus kamchatkensis* NASTPD13 isolated from Paudwar hot spring of Myagdi, Nepal, [10] and present its annotated draft genome. Additionally, we provide a comparative analysis of the GHs of strain NASTPD13 with four other sequenced *Anoxybacillus* spp. The current study describes the genomic level of thermophilic bacteria from the hot spring of Nepal and explores its cellular and molecular features in detail.

## 2. Materials and Methods

### 2.1. Isolation Details of NASTPD13

1 g of biomat sample collected from Paudwar hot spring [10] was suspended in 10 ml of nutrient broth (Himedia) and incubated at 60°C water bath, The diluted cultures (100 *μ*l) were spread on nutrient agar (HiMedia) plate and incubated at 60°C for 24 to 48 hours [11]. Single colony was picked, and the pure cultures were stored as glycerol stocks for further study.

### 2.2. Growth Conditions

*A. kamchatkensis* NASTPD13 was cultured on nutrient agar at 60°C for 18 h. Single colony was transferred into the nutrient broth and incubated at 60°C at 200 rpm for 18 h. Cells were harvested by centrifugation at 10,000 × g for 5 min using a Microfuge® 16 centrifuge (Beckman Coulter, Brea, CA, USA).

### 2.3. Biochemical Characterization

Apart from some manual biochemical characteristics of NASTPD13 explained in [12], Biolog MicroPlates technique was used for detail biochemical characteristics of NASTPD13. In Biolog MicroPlates technique, each well is coated with a single carbon source and after incubation of the culture shows the detail biochemical characteristics of individual bacteria in a single test. Biolog GN microplates (Biolog, Hayward, CA, USA) were used for NASTPD13. As per the manufacturer protocol, pure colonies of NASTPD13 from nutrient agar were suspended in GN/GP fluid to a specified density. 150 *μ*l of bacterial suspension was pipetted into each well, and the plates were incubated at 60°C up to 24 h. After incubation, plates were read in Biolog MicroStation™ system.

### 2.4. Antibiotic Susceptibility Testing

The antibiotic susceptibility test of NASTPD13 was performed by standard Kirby Bauer's disc diffusion tested as per CSLI guidelines [13]. The antibiotics tested were vancomycin, erythromycin, gentamycin, chloramphenicol, penicillin G, cefixime, rifampicin, kanamycin, azithromycin, novobiocin, lomefloxacin, and bacitracin. The antibiotic discs (6 mm diameter) were purchased from HiMedia Laboratory Ltd., Mumbai (India).

### 2.5. Transmission Electron Microscope (TEM)

NASTPD13 grown in minimum salt medium [10] containing 0.5% of beechwood xylan and for control in nutrient broth were individually harvested. Cells were centrifuged at 12,000 × g for 10 min. After centrifugation, the pellets were washed twice with PBS and then diluted with PBS to a cell density of 10^8^ CFU/ml. Washed cells were further stained with 0.1% (wt/vol) sodium phosphotungstate (PTA; Sigma, 32 USA) for 1 minute. Washed cells were negatively stained with 0.1% (wt/vol) sodium phosphotungstate (PTA; Sigma, USA) in water for 1 min on a carbon-coated copper grid (300 mesh; PolyScience cat# 71150). Grids were observed under JEOL JEM 2100, 200 kV transmission electron microscope (TEM) at a resolution of 0.1 to 0.2 m, and the TEM images were captured [14].

### 2.6. The Phylogenetic Analysis

NASTPD13 chromosomal DNA was extracted using the DNA Purification Kit (Promega Inc., Madison, WI, USA), and the 16S rRNA gene was amplified using universal primers 27f (5′-AGAGTTTGATCCTGGCTCAG-3′) and 1492r (5′-GGTTACCTTGTTACGACTT-3′). The amplified product was purified, using a QIA quick PCR purification kit (Qiagen Inc., San Diego, CA, USA), and sequenced in an ABI Prism 3700 automatic DNA sequencer by the use of a BigDye Terminator Cycle Sequencing kit (Applied Biosystems, Inc., Palo Alto, CA, USA). The sequence obtained was analyzed by the National Center for Biotechnology Information (NCBI) (http://www.ncbi.nlm.nih.gov/). The neighbor-joining phylogenetic tree was constructed with some species of the genus *Anoxybacillus* based on the 16S rDNA sequences by MEGA 6.0 [15].

### 2.7. Whole-Genome Sequencing, Assembly, and Annotation

Whole-genome sequence analysis of *Anoxybacillus kamchatkensis* NASTPD13 was done at CSIR-Institute of Microbial Technology, Chandigarh, India. Genomic DNA was extracted from the isolates using ZR Fungal*/*Bacterial DNA MiniPrep Kit (Zymo Research Corporation, Orange, CA, USA) as per the manufacturer's guidelines. Contaminations, quality, and concentration of genomic DNA were checked by using NanoDrop (Thermo Fisher Scientific, MA, USA), and quantification was done by Qubit 2.0 Fluorometer (Invitrogen, Carlsbad, CA, USA). Library preparation was done by using the Nextera XT sample preparation kit (Illumina, Inc., San Diego, CA, USA) with dual indexing adaptors as per the instruction manual. Sequencing of libraries was done on in-house Illumina MiSeq sequencing platform (Illumina, Inc., San Diego, CA, USA), in a 2 × 250 paired end run. Adapter trimming was done by MiSeq Control software (MCS). Quality of obtained reads was checked by using FastQC. Obtained reads were assembled using CLC Genomics Workbench v7 (CLC Bio-Qiagen, Aarhus, Denmark) with minimum contig length as 500 bp. 16S rRNA sequences were extracted from the assembled genomes using RNAmmer 1.2 server [16] and were characterized by Ez-taxon server [17], and tRNA was calculated by tRNAScan-SE ([18]) and CRISPRFinder [19] for finding CRISPR in the genome. The GHs were identified and verified using the dbCAN CAZy ([20]). Average nucleotide identity (ANI) was calculated by JSpecies [21], and Digital DNA-DNA hybridization (dDDH) was calculated by GGDC 2.0, DSMZ [22]. Assembled genomes were then annotated using Rapid Annotation using Subsystem Technology (RAST) [23] and BASys (a web server for automated bacterial genome annotation) [24]. BLAST Ring Image Generator (BRIG) software was used for comparison and visualization of genomes [25].

### 2.8. Comparative Genome Analysis

The comparative genomics helps to visualize the results and discovers correlations and trends in large datasets which makes understanding and interpretation of the data easier and generate figures for communicating results [26]. *Venn Painter* [26] and BLAST Ring Image Generator (BRIG) [25] were used for the comparative genome analysis of *A. kamchatkensis* NASTPD13.

## 3. Results and Discussions

### 3.1. Organism Information

**Figure 1:**
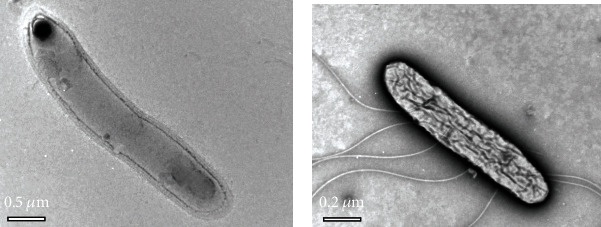
Transmission electron microscopy (TEM) micrograph of negatively stained cell of *A. kamchatkensis* NASTPD13. (a) Grown in nutrient broth. (b) Grown in minimal salt medium containing xylan as a carbon source.

**Figure 2:**
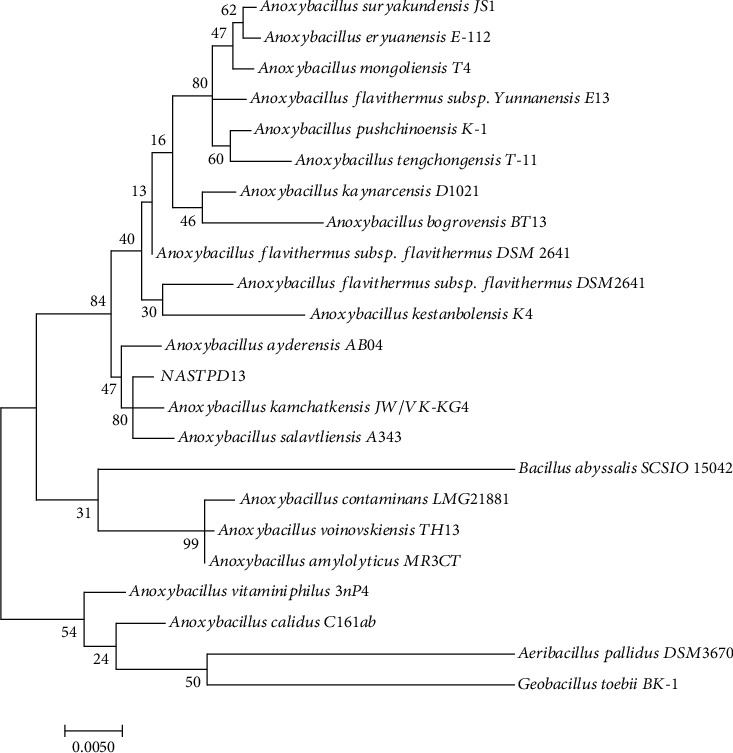
Phylogenetic trees of 16S rRNA gene sequences showing the relationship between NASTPD13 and representative *Anoxybacillus* sp. calculated with MEGA 6.0 software.

*Anoxybacillus kamchatkensis* NASTPD13 was isolated from Paudwar hot spring of Myagdi district, Nepal [27]. The colonies of NASTPD13 were 1–2 mm in diameter, cream-colored, and regular in shape with round edges. The strain was Gram-positive, rod-shaped containing spherical endospore, and catalase and oxidase positive and has ability to reduce nitrate to nitrite [12]. NASPD13 is a facultative anaerobe and moderately thermophilic that can grow at a wide temperature range of 37–70°C (optimum 55-60°C) and pH 5.0–11.0 (optimum pH 6.5–7.5) [12]. TEM image showed that cells were 0.7–0.4 *μ*m in size (Figure 1). NASTPD13 was able to utilize carbon sources including starch, gelatin, D-glucose, D-raffinose, D-sucrose, D-xylose, D-fructose, L-arabinose, maltose, and D-mannose, whereas there was no growth in the presence of vancomycin, erythromycin, gentamycin, chloramphenicol, penicillin G, cefixime, rifampicin, kanamycin, azithromycin, novobiocin, lomefloxacin, and bacitracin [10]. Phylogenetic analysis of 16S rRNA of NASTPD13 showed clusters together with *Anoxybacillus kamchatkensis* JW/VK-KG4 [9] (Figure 2).

### 3.2. TEM Imaging

The TEM image of NASTPD13 grown in nutrient broth (Figure 1(a)) showed rod-shaped (diameter 0.5 *μ*m) bacilli with no flagella, and the cell was surrounded by electron-transparent extracellular zone, and the cytoplasm was homogeneous with a terminal round shape endospore. In the presence of xylan, the cell diameter was 0.2 *μ*m with flagella (Figure 1(b)). An observable change of the electron-transparent material and a disorganized and distorted structure of cytoplasm were seen. Bacteria change their morphology depending on the carbon substrate used ([28]); phenotypic changes seen in NASTPD13 is due to the xylan present in the medium, and utilization of xylan explains the presence of xylan-degrading enzymes in NASTPD13. Various studies on the flagellar system of bacteria have reported that some bacteria have the unique property of switching on and off their flagellar system with some unknown mechanism for their survival in an unfavorable condition. Similar results have also been reported by [2] suggesting that *Anoxybacillus* spp. SK3-4 and DT3-1 were motile in certain media whereas nonmotile in certain growth media. Interestingly, this study also reports the TEM image of *Anoxybacillus kamchatkensis* NASTPD13 showing lots of flagella.

### 3.3. Whole-Genome Sequencing and Species Identification

The genome size of *A. kamchatkensis* NASTPD13 was 2,866,796 bp with GC 41.6% which assembled into 134 contigs with coverage of 184x. Genome features and assembly statistics of NASTPD13 are listed in Table 1. Species identification and phylogenetic relatedness are best studied by phylogenomic markers, average nucleotide identity (ANI) [29], and digital DNA-DNA hybridization (dDDH) [30].  Table 2 summarizes the pairwise ANI values of *Anoxybacillus* sp. *A. kamchatkensis* NASTPD13 with 14 other species. The whole-genome ANI value between *Anoxybacillus* sp. strain NASTPD13 and close relatives including *A. kamchatkensis* G10 [31], *A. gonenesis* G2 [32], *A. mongoliensis* MB4 [33], and *A. thermarum* AF/04 (T) [34] as 99.4%, 98.9%, 94.3%, and 93.6%, respectively. The dDDH of strain NASTPD13 neighbor strains was 95.1%, 85.5%, 73.9%, and 67.3%, respectively. *A. kamchatkensis* NASTPD13 showed the highest ANI of 99.4% and dDDH of 95.1% with *A. kamchatkensis* G10. The whole-genome shotgun project was deposited at DDBJ/EMBL/GenBank under the accession number NQLB000000000. The NCBI BioProject accession number is PRJNA397640, and the biosample accession number is SAMN07482658.

### 3.4. Annotation of NASTPD13

**Figure 3:**
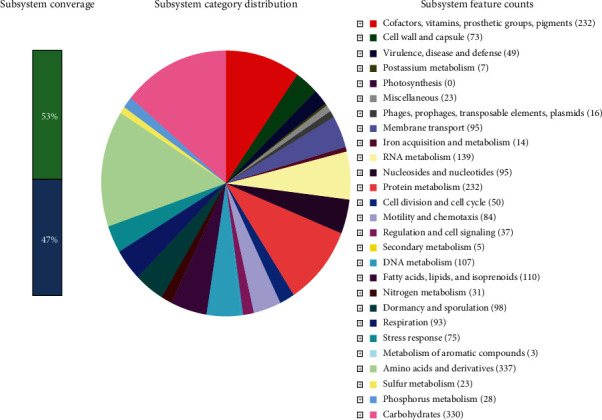
Distribution of major protein coding genes of *Anoxybacillus kamchatkensis* strain NASTPD13 annotated by Rapid Annotation System Technology (RAST) server. The 27 most abundant subsystem categories in strain NASTPD13 are explained by the pie chart. The bar explains about the subsystem coverage. The green color represents features that are found in RAST subsystem, and the blue color represents features not assigned to a subsystem.

**Figure 4:**
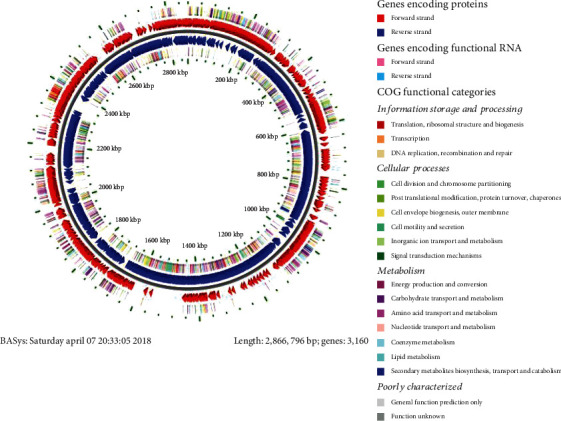
A graphical circular map of the *A*. *kamchatkensis* NASTPD13 genome by BASys (Bacterial Annotation System). From outside to the center: genes on the forward strand (colored by COG categories), genes on forward strand (red), genes on reverse strand (blue), and genes on the reverse strand (colored by COG categories).

**Figure 5:**
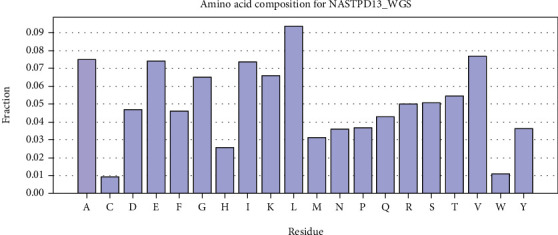
Amino acid compositions of *Anoxybacillus kamchatkensis* NASTPD13 by BASys. Amino acid composition values were extracted from the faa file obtained for this accession from NCBI. Each bar represents the fraction of the total amino acids matching the given residue.

RAST predicted 3169 genes among which 2992 were coding sequences (CDS) in the NASTPD13 genome. The subsystem distribution revealed a total of 436 subsystems among which amino acid and derivative subsystem featured with largest number 337 CDS. Other major subsystems annotated were carbohydrate 330, protein metabolism 232, cofactors, vitamins, prosthetic groups and pigments 232, RNA metabolism 139, sulfur metabolism 23, and motility or chemotaxis 84 (Figure 3). BASys annotated 3160 genes that were encoding various proteins and displayed in the form of circular DNA for easy representation of genomic data (Figure 4). The highest amino acid residue content was predicted for leucine followed by valine, alanine, glutamic acid, and isoleucine (Figure 5).

### 3.5. Comparative Analysis of NASTPD13

**Figure 6:**
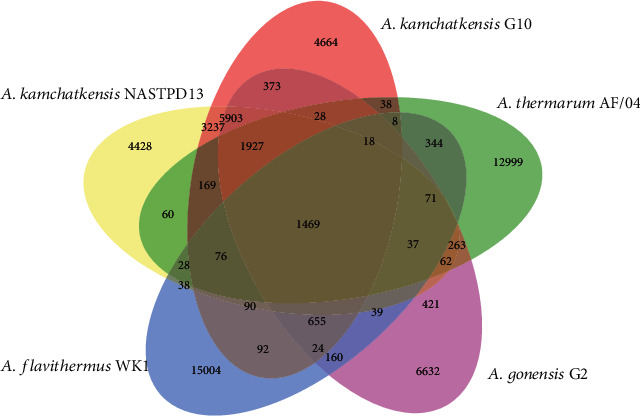
Venn diagram showing genes shared between four *Anoxybacillus* species. Isolates are denoted by colors: *A. kamchatkensis* NASTPD13 (yellow), *A. kamchatkensis* G10 (red), *A. thermarum* AF/04 (green), *A. gonensis* G2 (pink), and *A. flavithermus* (WK1).

NASTPD13 genome and other four *Anoxybacillus* spp. shared 1469 genes as shown in the Venn diagram (Figure 6). NASTPD13 and G10 shared 3237 CDS exclusively. In addition, 421, 60, and 38 genes are shared between NASTPD13 and G2, AF/04, and WK1, respectively, showing that NASTPD13 apart from G10 are more closely related to G2 than the other strains.

**Figure 7:**
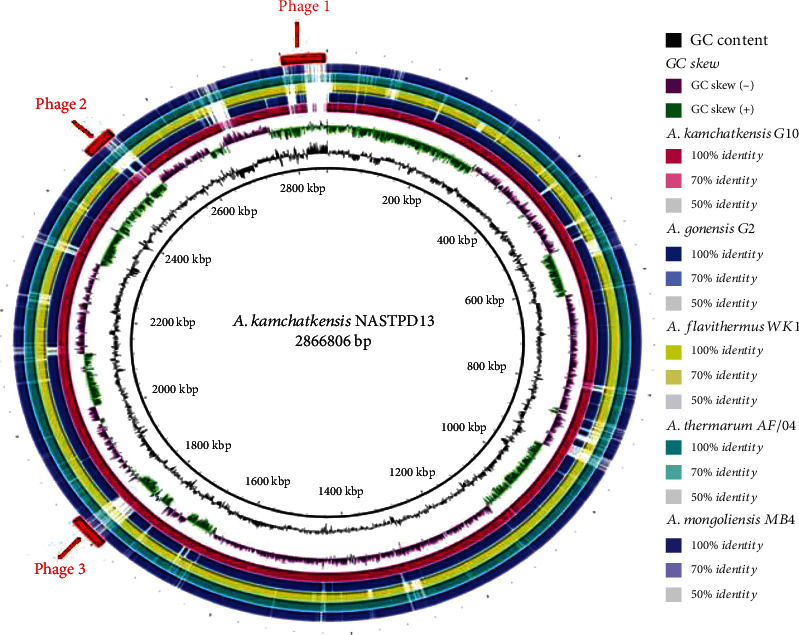
BRIG image showing whole genome comparison of *A. kamchatkensis* NASTPD13 with other members of genus *Anoxybacillus* spp. using BLAST Ring Image Generator.

Further, comparing the genome of G10, G2, MB4, and AF/04 (T) with NASTPD13 as a reference strain is sequenced in this study. The output image showed similarity between NASTPD13 and the other comparative four genomes as a set of concentric ring where BLAST matches are seen in different sliding colors defining the percentage of similarity among them. Even though small differences between the genome are not apparent in the BRIG concentric image, but it can be clearly seen in Figure 7 that *Anoxybacillus kamchatkensis* G10 genome is again the most similar to NASTPD13 among the strains analyzed.. The BRIG image also displays the bacteriophage genes in the NASTPD13 genome ranging in size between 33.7 kb and 64.8 kb. Phage 1 located at 1789826-1845786 bp, consisting of 229 ORFs, had the highest homology to phage Thermu_OH2_0127842 (28) of 33.7 kb; Phage 2 located at 2480822-2529216, consisting of 319 ORFs, had highest homology to Phage Bacter_Lily_NC_028841 (12) of 48.3 kb; and Phage 3 located at 2800662-2865493, consisting of 396 ORFs, had highest homology to Phage Entero_Lambda_NC_001416 (35) of 64.8 kb (Figure 7). Phage contributes to the evolution of their host bacteria by horizontal transfer of genes [35].

### 3.6. Analysis of the GHs in *A. kamchatkensis* NASTPD13 and Other *Anoxybacillus* Genomes

The photosynthetically fixed carbon in the plant cell wall is rich in organic carbon compound that can be converted to renewal biofuel. The degradation of plant polymers for various biotechnological uses requires combined action of several enzymes [36]. Glycoside hydrolase are potentially involved in the degradation of plant biomass [37]. The dbCAN CAZy database detected 21 genes in NASTPD13 genome encoding GH enzyme that belongs to GH families: 13, 23, 31, 32, 36, 42, 52, 65, and 109.The GHs are grouped based on their catalytic ability (Table 3). In NASTPD13, nine GH enzymes were active 275 on *α*-chain polysaccharides whereas five were specific for *β*-linked polysaccharides (i.e., cellulose and xylan). Along with GH families that hydrolyze the *β*-1,4-xyloside linkages of xylan, the CAZy database showed that NASTPD13 contains many other genes encoding for lignocellulose-degrading enzymes. The result showed 107 different CAZyme genes, encompassing all six CAZy families: 21 glycoside hydrolases (GHs), 21 glycosyltransferases (GTs), 15 carbohydrate esterases (CEs), 1 polysaccharide lyases (PLs), 4 auxiliary activities (AAs), and 23 carbohydrate-binding modules (CBMs). CBM (carbohydrate binding modules) families are capable of binding to xylan. The finding suggests that presences of various glycohydrolase genes in NASTPD13 hydrolyze the glycosidic bonds present in complex sugars.

### 3.7. Xylose Metabolism by *A. kamchatkensis* NASTPD13

Hemicellulosic sugars, especially d-xylose, are abundant in lignocellulosic biomass that can be recovered from by various physical and chemical pretreatments of the biomass, and also, D-xylose yields better from the biomass than d-glucose from cellulose. Xylose is one of the major fermentable sugars in nature after glucose; therefore, fermentation of xylose is necessary for producing biofuels, such as ethanol, from lignocellulosic biomass [38].

Hemicellulosic sugars has also been reported as a feedstock for production of ethanol and other chemicals but d-xylose is not so readily utilized as d-glucose due to the complex biochemical pathways for pentose and hexose metabolisms [39]. Various pathways have been employed by prokaryotes and eukaryotes for pentose assimilation [40].

**Figure 8:**
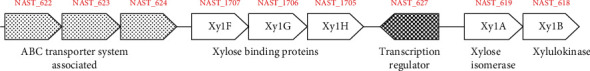
Genes encoding proteins for xylose metabolism located on the chromosome of *Anoxybacillus kamchatkensis* NASTPD13.

Thermophilic bacteria capable of fermenting both glucose and xylose are on high industrial demand (Yejun [41]). Thermophiles like *Anoxybacillus* sp. are relevant to the industry, because they produce thermozymes that match the harsh industrial process [2]. The observed ability of *A. kamchatkensis* NASTPD13 to utilize xylose in our previous study [10] developed our keen interest in the investigation of the xylose metabolism pathway and performed the whole-genome sequence of *A. kamchatkensis* NASTPD13. NASTPD13 genome revealed the presence of genes related to xylose metabolism such as xylose-binding protein xylG (NAST_1706), xylF (NAST_1707), xylH (NAST_1705), ABC transporter system-associated protein (NAST_622, NAST_623, NAST_624), transcription regulator (NAST_627), xylose isomerase (NAST_619) encoding gene xylA, and gene xylB coding for a xylulokinase (NAST_618) (Figure 8). XylA deals with isomerization of xylose to xylulose, while XylB convert xylulose to xylulose-5-phosphate. NASTPD13 xylan ABC transporter system and *β*-xylosidase continue to further step of xylose metabolism.

To date, in the family Bacillaceae, genome sequencing of *Geobacillus* (>80 projects) and *Bacillus* (>1500 projects) have been undertaken and have been registered in the NCBI BioProject database, whereas genomic studies on *Anoxybacillus* are rather limited (16 registered projects) [5]. These findings on *A. kamchatkensis* NASTPD13 genome add knowledge on the industrially important *Anoxybacillus* genomes. This study provides additional information on the xylose metabolism in *Anoxybacillus kamchatkensis* that can further help in the detailed study of the species.

## 4. Conclusion

*Anoxybacillus kamchatkensis* NASTPD13, isolated from Paudwar hot spring of Nepal, is Gram-positive with terminal endospore rod-shaped bacilli [10]. The TEM image of NASTPD13 grown in the xylan-containing medium showed major changes in their cell morphology along with the interesting property of flagella production in a certain growth medium such as xylan. The genome size of NASTPD13 is 2.8 Mb. Comparison of the NASTPD13 genome with the closely related strain of *Anoxybacillus* spp. against various databases showed the closed relationship with *Anoxybacillus kamchatkensis* G10. Previous study explained in [10] related to NASTPD13 xylanase and combining the data from this study related to presence of genes related to metabolism of Xylan showed the presence of xylanase and *β* xylosidase gene that are essential for complete degradation of xylan to pentose sugar xylose. The gene related to utilization of xylose (xylG, xylF, and xylH) is also present in *Anoxybacillus kamchatkensis* NASTPD13. The presence of all the genes related to the metabolism of xylan in *Anoxybacillus kamchatkensis* NASTPD13 genome emphasizes its industrial importance along with promising sources of lignocellulase in second-generation biofuel production.

## Figures and Tables

**Table 1:** General features and comparison of genomes of NASTPD13 with other *Anoxybacillus* genome.

Attribute	NASTPD13	*A. gonensis* G2	*A. mongoliensis* MB4	*A. kamchatkensis* G10	*A. thermarum* AF/04
Genome size (bp)	2,866,796	2,803,668	2,807,516	2,858,657	2,736,908
G+C content (%)	41.6	41.7	41.7	41.3	42.0
N50	103,759	2,803,668	78,095	130,036	37,018
No. of contigs	134	1	93	65	159
Coding sequence	2992	2808	2798	2910	3004
Pseudogenes	75	56	129	107	—
Genes (RNA)	102	106	113	69	117
rRna	19	24	32	9	9
tRna	79	78	77	56	74
ncRNA	4	4	4	4	1
CRISPR arrays	1	2	1	1	2
Accession number	NQLB000000000	JRZG01000000	MRZM00000000	ALJT00000000	JXTH00000000

**Table 2:** Genomic comparison of *A. kamchatkensis* NASTPD13 and 14 other sequenced *Anoxybacillus* spp. using ANI.

	NASTPD13	AB04	AF/04	BAA-2555	BC01	DSM15939	DSM27374	G2	G10	K1	P3H1B	PS2	MB4	SK3-4	WK1
NASTPD13	100	94.29	93.6	72.8	94.2	74.9	87.0	98.9	99.4	85.5	72.7	72.9	94.3	93.7	88.2
AB04	94.3	100	94.4	73.1	97.3	75.2	87.4	94.2	94.1	86.4	72.7	72.9	97.2	97.4	88.0
AF/04	93.7	94.6	100	73.9	93.9	74.8	87.1	93.6	93.5	86.6	72.7	73.0	93.9	94.1	88.0
BAA-2555	71.6	71.8	71.9	100	71.8	73.9	71.2	71.6	71.6	71.9	88.2	75.2	71.8	71.8	71.8
BC01	94.2	97.4	98.9	73.0	100	74.9	87.2	93.9	94.0	86.2	72.5	72.8	96.5	96.8	87.7
DSM15939	74.8	75.0	74.8	75.8	74.8	100	74.2	74.6	74.9	74.4	75.9	76.9	75.0	74.9	75.1
DSM27374	87.1	87.4	87.1	72.3	87.3	74.0	100	87.2	87.0	85.9	72.3	72.6	88.2	87.4	88.4
G2	98.8	94.3	93.7	72.8	94.2	74.5	87.3	100	98.7	85.9	72.6	72.8	94.4	94.0	88.4
G10	**99.4**	94.2	93.5	72.8	94.2	74.8	87.0	98.7	100	85.5	72.8	72.9	94.3	93.7	88.1
K1	85.5	86.4	86.3	73.2	86.3	74.6	86.0	85.8	85.6	100	72.3	72.6	86.0	86.4	86.4
P3H1B	72.4	72.3	72.5	98.1	72.2	75.4	72.2	72.2	72.5	72.3	100	78.2	72.2	72.4	72.4
PS2	72.7	72.6	72.8	77.8	72.7	76.6	72.4	72.7	72.7	72.6	78.3	100	72.7	72.6	72.7
MB4	94.2	97.2	93.6	72.9	96.5	75.0	88.2	94.1	94.1	86.0	72.7	72.9	100	96.6	88.0
SK3-4	93.6	97.5	94.0	73.2	96.8	74.6	87.4	93.8	93.5	86.4	72.6	72.9	96.7	100	88.1
WK1	88.3	87.9	87.8	73.0	87.7	75.3	88.4	88.2	88.2	86.3	73.0	72.9	88.0	8.1	100

The reference protein sequence is denoted as 100%. AB04: *A. ayderensis* AB04 [5]; AF/04: *A. thermarum* AF/04 [42]; BAA-2555: *Anoxybacillus geothermalis* [43]; BCO1: *Anoxybacillus* sp. BCO1 [44]; DSM15939: *Anoxybacillus amylolyticus* DSM15939 [42]; DSM27374: *Anoxybacillus suryakundensis* strain DSM 27374 [45]; G2: *A. gonensis* G2 [32]; G10: *A*. *kamchatkensis* G10 [31]; K1: *Anoxybacillus pushchinoensis* K1 [46]; P3H1B: *Anoxybacillus* sp. P3H1B [47]; PS2: *A. tepidamans* PS2 [48]; MB4: *Anoxybacillus mongoliensis* MB4 [33]; SK3-4: *Anoxybacillus* sp. SK3-4 [49]; WK1: *A. flavithermus* WK1 [4].

**Table 3:** Glycoside hydrolases (GHs) identified in *A. kamchatkensis* NASTPD13 and comparing NASTPD13 with other reported *Anoxybacillus* genomes GHs.

GHs	Enzymes	NASTPD13	G10	MB4	G2	AF/04
13	1,4-Alpha-glucan branching enzyme	100.00	100.00	99.76	99.54	96.14
13	Oligo-1,6-glucosidase	100.00	100.00	96.76	99.54	96.14
13	Trehalose-6-phosphate hydrolase	100.00	100.00	98.38	99.10	98.74
13	Alpha-glucosidase	100.00	99.82	99.10	99.82	98.92
13	Cytoplasmic alpha-amylase	100.00	100.00	93.96	—	—
13	Pullulanase	100.00	100.00	95.74	99.32	93.19
13	Maltodextrin glucosidase	100.00	99.45	—	98.39	—
23	Murein hydrolase	100.00	100.00	—	97.04	93.10
31	Alpha-glucosidase	100.00	99.82	99.10	99.82	98.92
32	Sucrose-6-phosphate hydrolase	100.00	100.00	97.99	97.99	91.92
36	Alpha-galactosidase	100.00	100.00	—	55.13	—
42	Beta-galactosidase	100.00	100.00	—	99.69	—
52	Beta-xylosidase	100.00	100.00	89.08	99.43	—
65	Maltose phosphorylase	100.00	100.00	96.41	99.87	—
109	Oxidoreductase	100.00	100.00	90.99	99.70	—

^∗^NASTPD13: *A. kamchatkensis* NASTPD13; G10: *A. kamchatkensis* G10; MB4: *A. mongoliensis* MB4; G2: *A. gonensis* G2; AF/04: *A. thermarum* AF/04.

## Data Availability

*Anoxybacillus kamchatkensis* NASTPD13 whole-genome sequence was deposited in DDBJ/EMBL/GenBank under the accession number NQLB000000000. The NCBI BioProject accession number is PRJNA397640, and the biosample accession number is SAMN07482658.
